# Micronutrient status of individuals with overweight and obesity following 3 months’ supplementation with PolyGlycopleX (PGX®) or psyllium: a randomized controlled trial

**DOI:** 10.1186/s40795-022-00534-7

**Published:** 2022-05-03

**Authors:** Sebely Pal, Jenny McKay, Suleen Ho, Monica Jane, Roland J. Gahler, Simon Wood

**Affiliations:** 1 School of Public Health, Faculty of Health Sciences, Curtin University, Perth, WA Australia; 2 Factors Group of Nutritional Products Inc. R & D, Burnaby, BC Canada; 3 InovoBiologic Inc, Calgary, AB Canada; 4 Faculty of Land and Food Systems, University of British Columbia, Vancouver, BC Canada

**Keywords:** Obesity, Dietary fibre, Psyllium, PGX®, Micronutrients, Absorption, Bioavailability, Deficiency

## Abstract

**Background:**

Safe and effective weight control strategies are needed to curtail the current obesity epidemic worldwide. Increasing dietary fibre has shown positive results with weight loss as well as in the reduction of metabolic syndrome risk factors. However, fibre can act as an inhibitor to the bioavailability of micronutrients in the gastrointestinal tract. While there is a substantial amount of scientific research into psyllium fibre, PolyGlycopleX (PGX®) is a novel fibre and as yet the effects of PGX® on micronutrient status is not well researched.

**Aim:**

To determine whether 3-months’ supplementation with 15 g of psyllium or PGX® fibre daily affects micronutrient status of overweight and obese adults.

**Methods:**

Overweight and obese individuals with a BMI between 25–40 kg/m^2^ and aged between 18 and 65 years, but otherwise healthy, were instructed to consume a 5 g sachet of psyllium, PGX® fibre or a rice flour placebo three times a day for 52 weeks as part of a larger long-term study. Blood sample data for the first 3 months were analysed for associations between serum micronutrient levels and psyllium fibre and/or PGX® supplements.

**Results:**

No significant differences between fibre supplement groups and micronutrient status were found after 3 months at *p* > 0.05. Dietary intake of vitamin C was significantly lower for PGX® at 3 months compared to baseline and compared to control (*p* < 0.05). Folate was significantly lower in the control group after 3 months (*p* < 0.05). In the psyllium group, folate, sodium, zinc and magnesium intake decreased after 3 months (*p* < 0.05). A limitation of dietary intake data (tertiary measure) is the potential for inaccurate self-reporting, although reduced nutrient intake could be due to the satiating effect of dietary fibre.

**Conclusions:**

There were no significant between group differences in serum micronutrient concentrations after a 3-month psyllium fibre or PGX® supplementation intervention of 15 g per day. Fibre supplementation is unlikely to compromise the nutritional status of overweight and obese individuals in the short term. Further research is recommended to monitor micronutrient status over a longer period or with a higher fibre dosage.

**Supplementary Information:**

The online version contains supplementary material available at 10.1186/s40795-022-00534-7.

## Background

Obesity is the result of both genetic and environmental influences [1]. The habitual consumption of more than the required amount of total energy, through more processed or of low nutritional density foods, is said to contribute to excess weight [2] and may lead to a low micronutrient status [3]. Micronutrient deficiency can be defined as a prolonged lack of essential vitamins and minerals required for proper growth and development or maintenance of optimal health [4]. Overweight or obesity may negatively impact the bioavailability and utilisation of micronutrients by interfering with such processes as absorption, excretion, storage/distribution (eg. fat sequestering, tissue dispersion), or metabolism (eg. catabolic losses, possibly oxidative) [5]. Increased physiologic requirements, and/or lower absolute total dietary intake may impair the micronutrient status of adults with overweight and obesity [6].

One food component that is commonly lacking in the diet of overweight or obese individuals is dietary fibre. The benefits of dietary fibre are well known, however most people find it difficult to eat the required amounts of fibre through increased fruit and vegetable intake, as well as cereals and grains as shown in the Australian Health Survey: Consumption of food groups from the Australian Dietary Guidelines 2011–2012 [7]. Present estimations of dietary fibre intake in Australian, Canadian, European and American adults is approximately 15–25 g/day, which is below the current recommendations for adults of 25–30 g/day in these regions [8, 9]. Research has consistently shown that those who routinely consume foods high in dietary fibre are less likely to be overweight or obese than those with a low fibre diet, as fibre promotes satiety [10], and can lead to reduced energy intake [11–13].

Fibre supplements may provide a cost effective and easy alternative for increasing the fibre content of a persons’ diet without the need for other major dietary modifications. However, high intakes of certain dietary fibre sources have been linked to deficiencies of calcium, iron, trace metals, and vitamin D and E due to its role in the food matrix and metabolism [14]. The most common fibre promoted for health benefits is psyllium. Psyllium is widely consumed in Metamucil®, a commercially available fibre product that is marketed as a tool to promote bowel regularity and general wellbeing. Psyllium is not fermented [15] and does not increase gas or flatulence [16–20], making it well tolerated and accepted [21]. Psyllium studies have found beneficial effects on glucose and insulin homeostasis, lipids and lipoprotein, body weight, body composition and appetite [10].

PolyGlycopleX (PGX®) is another soluble fibre supplement that is showing positive health benefits. PGX® is a novel, highly viscous functional non-starch polysaccharide complex, with developing viscosity, manufactured by a proprietary process (EnviroSimplex®). PolyGlycopleX® is 3–5 times more viscous than any known individual polysaccharide [22] and is a compound of 3 different natural fibres: konjac (glucomannan), sodium alginate and xanthan gum [13].

Recent research has shown that adding 2.5–5 g of PGX® to a meal is highly effective in reducing postprandial glycaemia, lowering the glycaemic index of food [23] and modifying satiety hormones [13].

Studies looking at fibre supplementation have proven that increasing dietary fibre can convey a multitude of health benefits [10]. This is especially true for overweight or obese individuals attempting weight loss, as high dietary fibre supplementation has been correlated with weight, BMI and waist circumference reduction [24, 25]; however little is known regarding the effect this strategy has on the micronutrient status in this already vulnerable group. The biochemical effect of therapeutic doses of fibre in supplement form for weight loss need to be evaluated, as a high intermittent dosage may impair absorption of micronutrients in the gut, especially if taken prior to or during a meal [14]. This study aims to investigate the effect of 15 g daily supplementation with two fibre types, PGX® and psyllium, on micronutrient status when added to the habitual diet of overweight and obese Australian adults over three months. It was hypothesised that the absorption of some micronutrients may be compromised with the increase in daily fibre supplementation in the food matrix.

## Methods

### Participants

**Fig. 1:**
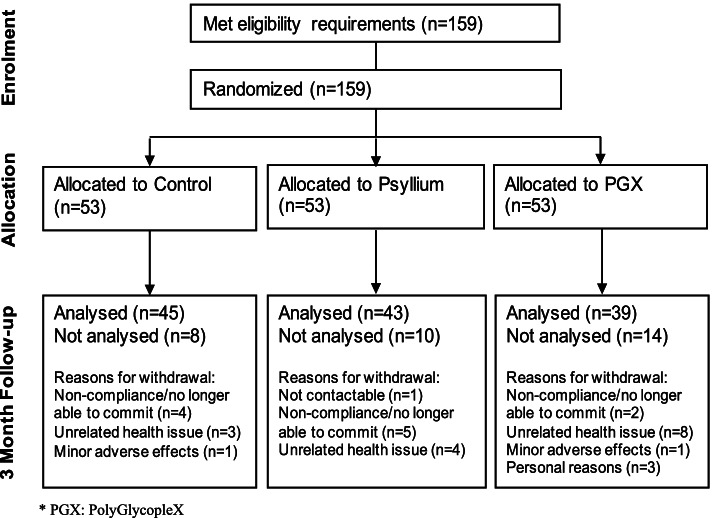
Participant Flow Diagram

Overweight and obese individuals (*n* = 159) aged between 18 and 65 years and with a body mass index (BMI) between 25–40 kg/m^2^, were recruited from the community in Perth, Australia. (See Fig. 1) Advertisements for the study were made via newspapers, flyers posted around Curtin University and local noticeboards, as well as radio advertising on Curtin FM. Individuals were screened either by telephone or online via Qualtrics using a questionnaire. Eligible participants attended an orientation session at Curtin University to assess suitability for the study, at which time the study details were also explained. Successful applicants were those who did not smoke, or take any medications that were lipid lowering, use steroids or other agents that may influence lipid metabolism. Those who took blood pressure medications were also excluded as were those with Type 1 or 2 diabetes mellitus, hypo and hyperthyroidism, or had any cardiovascular events within the last 6 months. The remaining exclusion criteria included, psychological unsuitability, major systemic diseases, gastrointestinal problems, proteinuria, liver, renal failure, any significant weight fluctuations over the past 6 months, vegetarianism or veganism and participation in any other clinical trials within the last 6 months. This study adheres to CONSORT guidelines and was approved by and conducted in accordance with the ethical standards of Curtin Human Research Ethics Committee. Data for this study was pulled from a clinical trial registered with the Australian New Zealand Clinical Trial Registry on 20/04/2011, registration number: ACTRN12611000415909.

### Study design

Serum micronutrient and dietary intake data was assessed at baseline and at 3 months for this study. The study was a randomised, controlled, double blind, parallel design intervention. The trial sponsors used the website (http://www.randomization.com) to ensure block randomisation to one of three groups (three randomly permuted blocks); The control group who consumed a placebo (which consisted of rice flour); the psyllium fibre supplement group; or the PGX® fibre supplement group. The supplement packages (control, PGX and psyllium) were identical in appearance and were provided by the manufacturer via single batch. This ensured the research assistants and participants were both blinded to the type of supplement being consumed. Assignment to a group was random and facilitated by research assistants at Curtin University after participant screening. Individual packages were marked by the participant ID and consisted of 5 g doses of the control, PGX or psyllium, with the group allocation only known to the trial sponsors to ensure blinding. ‘Quantities of rice flour and psyllium were determined by input, with the amounts weighed and checked by the dispensing and blending department while PGX was analysed according to USP Monograph FCC9 3rd Sup 2015’ [26, 27]. All identifiable information from participants was coded to ensure privacy. All requirements and instructions for the study were identical for each of the three groups and participants consumed a supplement in addition to their habitual diet. The control (rice flour) had a low energy and fibre content and was similar in texture and appearance to the fibre supplements, so provided an appropriate placebo. Participants were advised to consume 5 g supplement sachet containing either the psyllium, PGX® fibre or placebo, mixed with a minimum of 250 mL water 5—10 min before breakfast, lunch and dinner (15 g total) every day. Participants were asked to record any other supplements taken, such as vitamin, mineral or other for the duration of the study. All identifiable information from participants was coded and kept strictly confidential. Those who missed consecutive days of supplement consumption were discontinued from the trial.

### Data collection

Participants attended a briefing session on how to consume the supplements, complete the paperwork and comply with the study protocol. Dietary intake over the course of the trial was monitored through the completion of 3-day food diaries at each clinical visit. Participants in all groups were asked to maintain their usual diet for the duration of the study. To monitor compliance, all participants were required to complete a diary to record their supplement consumption and asked to return the empty and unused sachets of the supplements at their clinical visits. These sachets were then counted and recorded. The trial was conducted in compliance with the study protocol. All participants were asked to report any adverse events to the investigators immediately. Medical support was to be provided for any serious adverse events resulting from this study. In addition, such events were to be recorded by the Investigator and reported to HREC. Participants would then be given the option to withdraw from the study and all data and blood samples for this participant would be destroyed after withdrawal.

## Assessments

### Micronutrients blood analysis

Fasting blood samples drawn by venepuncture were collected at baseline (week 0) and week 12. Samples were collected into lithium heparin or serum separator tubes (5 ml) for antioxidant/vitamins A, C, D B_9_, B_12_ and E and minerals (calcium, iron, iodine, folate, magnesium, potassium, sodium and zinc) and then centrifuged at 2,500 rpm at 4ºC for 10 min using an Eppendorf centrifuge and prepared for storage at -80°C. Vitamins A, E, D, as well as vitamin B_9_, and B_12_ and thyroglobulin were measured using Enzyme-Linked Immunosorbent Assay kits (ELISA). ELISA combines antibody binding with enzymatic detection to identify molecules of interest; the result is a colour change that is measured by spectrophotometry at a particular wavelength [9]. Vitamin C and total antioxidant capacity was measured by colorimetric assay. All trace metals (calcium, magnesium, iron, zinc, sodium and potassium) were analysed by Flame Atomic Absorption Spectroscopy (FAAS).

### Three-day food diaries

Prior to commencement of the study, each participant attended a briefing session, which included instructions for completing the weighted 3-day food diaries. Completed food diaries were collected at the baseline and week 12 clinic appointments. Participants were required to record all food and drink consumed including water for three consecutive days. As much detail as possible was encouraged, including portion sizes, weights and brands. Dietary micronutrient data from the food diaries was extracted and analysed using Food Works Version 7 (Xyris Software, 2012). Participants were required to complete the food diaries as a reference, as it was necessary to make sure that any changes to serum micronutrients were not as a result of changes in dietary intake, as participants were instructed not to alter their ‘normal diet’.

### Outcome measures

The present study was designed to assess the nutritional status of overweight and obese Australian adults, via comparison of serum micronutrients and food diary analysis at baseline and week 12 in relation to: vitamins A, C, D, E, B_9_ and B_12_ and minerals calcium, iron, magnesium, potassium, sodium and zinc.

### Statistical analysis

A sample size of 24 subjects per group is predicted to provide sufficient power (80%) to detect a 3% difference in weight before and after treatment within a group. Recruiting a total of 50 subjects per group will accommodate for 50% dropouts. Calculations are based on an average mean weight of 80 kg and a standard deviation of 5% within a group on all eligible subjects. Per-Protocol statistical analysis was conducted using analysis of covariance to determine between groups differences in micronutrients for the two intervention groups, compared to the control group at 3 months (using the baseline values as covariate). Post hoc analysis was conducted using the Least Significant Difference method. Statistical significance was considered at *p* < 0.05. All analysis was conducted using SPSS 23.0 (IBM® SPSS® Statistics, New York, NY.

## Results

### Participants

There were 53 participants in each group (control, psyllium and PGX®) at the start of the study, at the 3 month mark the control group had 45 participants, the psyllium group had 43 participants and the PGX® group had 39 participants. Reasons for truancy were noncompliance/unable to commit, unrelated health issues, minor adverse effects (abdominal bloating or constipation/diarrhea) or personal reasons. There was no serious adverse effects reported.

### Baseline characteristics

Baseline between group values for serum and self-recorded dietary intake data are shown in Table 1. While participants in the psyllium fibre group showed slightly higher serum values at baseline for most micronutrients compared to PGX®, there were no significant between group differences (Control, Psyllium and PGX®) in serum micronutrient values as seen in Table 1. Interestingly, self-reported zinc intake from dietary intake data was found to be significantly lower in the PGX® group compared to control (11.5 ± 0.6 mg vs 14.1 ± 0.8, *p* = 0.030).

**Table 1:** Baseline participant characteristics

	**CONTROL**		**PSYLLIUM**		**PGX**	
	**Mean ± SEM**	**n**	**Mean ± SEM**	**n**	**Mean ± SEM**	**n**
Age (years)	49.8 ± 1.8	45	49.9 ± 1.7	43	47.9 ± 1.9	39
Weight (kg)	94.7 ± 2.5	45	91.2 ± 2.2	43	96.2 ± 2.9	39
BMI (kg/m^2^)	32 ± 0.6	45	31.7 ± 0.5	43	33.2 ± 0.7	39
Serum:						
Vitamin E (µg/mL)	7.4 ± 0.7	45	8.2 ± 0.9	42	7.8 ± 0.9	39
Vitamin B12 (pg/mL)	731.5 ± 69.7	44	755.4 ± 72.7	43	677.3 ± 73.1	39
Vitamin C (mg/dL)	63.9 ± 4.6	45	65.8 ± 4.0	43	63.6 ± 4.1	39
Vitamin A (ng/mL)	52.4 ± 3.0	45	49.5 ± 2.9	43	48.9 ± 3.1	39
Vitamin D (ng/mL)	10.7 ± 1.1	45	11.9 ± 1.1	43	9.9 ± 1.0	39
Folate (pg/mL)	2558.3 ± 370.6	45	2650.5 ± 333.2	42	2191.3 ± 333.8	38
Thyroglobulin (ng/mL)	10.2 ± 3.4	41	8.4 ± 0.9	41	7.6 ± 1.7	37
Potassium (µg/mL)	99.8 ± 1.7	45	98.6 ± 1.5	43	98.5 ± 1.7	39
Sodium (µg/mL)	2706.4 ± 29.1	45	2730.7 ± 40.3	43	2760.8 ± 41.1	39
Iron (µg/mL)	1.1 ± 0.1	45	1.0 ± 0.1	43	1.0 ± 0.1	39
Zinc (µg/mL)	0.3 ± 0.0	45	0.3 ± 0.0	43	0.3 ± 0.0	39
Calcium (µg/mL)	33.9 ± 1.4	45	35.3 ± 1.5	43	33.5 ± 1.2	39
Magnesium (µg/mL)	6.7 ± 0.2	45	6.9 ± 0.2	43	6.7 ± 0.1	39
Dietary intake:						
Vitamin E (mg)	9.1 ± 0.8	44	8.8 ± 0.7	43	8.6 ± 0.7	39
Vitamin C (mg)	109.1 ± 13.2	44	102.9 ± 12.7	43	97.1 ± 9.5	39
Retinol (µg)	365.4 ± 32.2	44	357.5 ± 46.3	43	385.7 ± 45.5	39
Beta carotene (µg)	2840.3 ± 415.4	44	3087.4 ± 346.8	43	2054.8 ± 182.3	39
Vitamin D (µg)	3.8 ± 0.5	44	3.5 ± 0.3	43	3.7 ± 0.4	39
Folate (µg)	398.1 ± 32.8	44	410.4 ± 31.4	43	304.1 ± 17.3	39
Iodine (µg)	132 ± 7.6	44	128.9 ± 7.3	43	123.9 ± 9.3	39
Potassium (mg)	3196.0 ± 118.6	44	3167.2 ± 136.7	43	2902.3 ± 120.9	39
Sodium (mg)	2961.1 ± 174.6	44	2613.8 ± 181.8	43	2669.7 ± 158.1	39
Iron (mg)	13.4 ± 0.8	44	12.1 ± 0.6	43	11.8 ± 0.6	39
Zinc (mg)	14.1 ± 0.8	44	13.6 ± 0.9	43	11.5 ± 0.6*	39
Calcium (mg)	907.1 ± 59.3	44	901.8 ± 48.8	43	882.0 ± 73.1	39
Magnesium (mg)	387.7 ± 20.3	44	373.7 ± 19.0	43	357.0 ± 19.7	39

^*^Significant compared to Control at *p* < 0.05. Between-groups difference was examined using one-way ANOVA

### Serum micronutrient values following 3 months fibre supplementation

Table 2 illustrates that there was no significant between group differences in serum micronutrient status after three months’ fibre supplementation. However, serum values for key micronutrients did increase within group from baseline for both fibre groups following supplementation when comparing Table 1 and 2. Folate levels for psyllium went from 2650.5 ± 333.2 pg/mL at baseline (Table 1) to 2833.3 ± 476.2 pg/mL at 3 months (Table 2) which is an increase of 6.9%. For PGX®, baseline levels were 2191.3 ± 333.8 pg/mL and 2417.9 ± 415.0 pg/mL after 3 months, which translates to a 10.3% increase. Sodium intake was found to be 11.8% higher following fibre supplementation in both fibre groups with baseline psyllium values of 2730.7 ± 40.3 µg/mL compared to 3051.6 ± 29.8 µg/mL at 3 months. PGX® baseline levels for sodium were 2760.8 ± 41.1 µg/mL and increased to 3040.5 ± 26.2 µg/mL (10.5%). Potassium levels were also 22.2% lower after supplementation with baseline psyllium group levels of 98.6 ± 1.5 µg/mL and 76.7 ± 3.9 µg/mL after 3 months. Similarly, PGX® baseline potassium decreased 28.8% with initial values of 98.5 ± 1.7 µg/mL and decreasing to 70.1 ± 3.8 g/mL at 3 months.

**Table 2:** Between group serum differences in nutritional status after three months of Psyllium or PGX fibre supplementation

	**CONTROL**			**PSYLLIUM**			**PGX**		
	**Mean ± SEM**	**Difference**	**n**	**Mean ± SEM**	**Difference**	**n**	**Mean ± SEM**	**Difference**	**n**
Vitamin E (µg/mL)	6.8 ± 0.6	-0.6	45	8.8 ± 1.0	0.6	43	7.8 ± 0.9	0	39
Vitamin B12 (pg/mL)	688.8 ± 65.6	-42.7	44	757.3 ± 72.7	1.9	43	688.9 ± 65.1	11.6	39
Vitamin C (mg/dL)	62.3 ± 3.4	-1.6	45	60.8 ± 3.2	-4.9	43	57.7 ± 2.8	-5.9	38
Vitamin A (ng/mL)	48.7 ± 3.0	-3.7	44	46.9 ± 3.2	-2.6	43	49.5 ± 3.2	0.6	39
Vitamin D (ng/mL)	10.0 ± 1.4	-0.7	45	10.3 ± 1.0	-1.6	43	8.6 ± 0.8	-1.3	39
Folate (pg/mL)	2822.9 ± 518.8	264.7	45	2833.3 ± 476.2	182.8	41	2417.9 ± 415.0	226.6	38
Thyroglobulin (ng/mL)	11.2 ± 3.4	1	42	8.5 ± 1.0	0.1	42	7.2 ± 1.2	-0.4	38
Potassium (µg/mL)	71.2 ± 3.5	-28.6	45	76.7 ± 3.9	-21.8	43	70.1 ± 3.8	-28.4	39
Sodium (µg/mL)	3012.4 ± 28.4	306	45	3051.6 ± 29.8	320.9	43	3040.5 ± 26.2	279.6	39
Iron (µg/mL)	0.8 ± 0.0	-0.2	45	0.8 ± 0.0	-0.3	43	0.9 ± 0.1	-0.1	39
Zinc (µg/mL)	0.5 ± 0.0	0.2	45	0.5 ± 0.0	0.2	43	0.5 ± 0.0	0.2	39
Calcium (µg/mL)	43.9 ± 0.7	9.9	45	44.2 ± 0.7	8.9	43	43.2 ± 0.7	9.7	39
Magnesium (µg/mL)	7.2 ± 0.1	0.5	45	7.4 ± 0.1	0.5	43	7.3 ± 0.2	0.5	39

^*^There were no significant between group differences *p* > 0.05. Between-groups difference was examined using one-way ANOVA

### Nutrient intake data following 3 months of fibre supplementation

Self-reported micronutrient intake values from the 3-day food diary for the three groups are shown in Table 3. Baseline values for dietary intake data were used as a covariate for between group differences (a) and the control was used as covariate for within group differences (b) (refer to Table 3). For vitamin C, a significant difference was found compared to baseline and compared to control for PGX® at *p* < 0.05 with a mean ± SEM of 102.8 ± 10.5 mg (baseline) and 69.5 ± 5.9 mg and a reduction in recorded intake of 30.276 mg. Folate also showed a significant reduction compared to baseline at *p* < 0.05 for the control group and the psyllium group. The control group decreased from 414.0 ± 35.6 µg at baseline to 340.3 ± 24.5 µg at week 12 (-71.103 µg). The psyllium group for dietary intake of folate went from 406.4 ± 32.8 µg to 340.7 ± 22.4 µg after 3 months fibre supplementation (-65.642 µg). Sodium was found to be statistically significant at *p* < 0.05 in the psyllium group compared to the control at *p* < 0.05, with 2615.4 ± 188.0 mg recorded at baseline and decreasing to 2334.8 ± 128.5 mg at 3 months (-280.569 mg). Zinc showed a significant reduction over 3 months fibre supplementation compared to baseline in the psyllium group with 13.6 ± 1.0 mg at baseline and 11.8 ± 0.7 mg at the 12 week mark (-1.857 mg). Dietary intake of magnesium also decreased significantly in the psyllium group by 37.584 mg from baseline (371.9 ± 19.9 mg) to 3 months (334.3 ± 15.8 mg). Overall there appeared to be a downward trend in dietary intake data of most micronutrients in both the psyllium and PGX® group compared to baseline and compared to the control group after 3 months fibre supplementation. However only the afore mentioned micronutrients were significant at *p* < 0.05. For example, iodine levels at 3 months for the PGX® group was lower at 118.1 ± 7.6 µg compared to the control group 138.3 ± 9.2 µg but not significant at *p* < 0.05. Calcium was also lower in the PGX® group at 3 months (754.0 ± 57.3 mg) compared to baseline (834.3 ± 60.6 mg), but again no significance at *p* < 0.05 was found.

**Table 3:** Within and between group differences in dietary micronutrient levels from baseline to 3 months of psyllium or PGX fibre supplementation

	Baseline	n	12 Weeks	Difference	n
**Vitamin E (mg)**					
Control	9.5 ± 0.9	39	8.5 ± 0.6	-1.033	40
Psyllium	8.8 ± 0.7	41	7.7 ± 0.5	-1.100	41
PGX	8.6 ± 0.8	33	8.5 ± 0.7	-0.137	32
**Vitamin C (mg)**					
Control	102.4 ± 9.7	39	115.4 ± 17.1	14.367	40
Psyllium	98.7 ± 12.0	41	104.9 ± 11.5	6.192	41
PGX	102.8 ± 10.5	33	69.5 ± 5.9^a,b^	-30.276	32
**Retinol (µg)**					
Control	394.8 ± 33.4	39	358.5 ± 25.7	-33.701	40
Psyllium	352.3 ± 48.3	41	3356.0 ± 460.2	-47.459	41
PGX	363.9 ± 44.6	33	2818.2 ± 515.0	1410.918	32
**B Carotene (µg)**					
Control	2893.6 ± 453.9	39	2971.3 ± 345.9	143.611	40
Psyllium	3044.8 ± 349.2	41	3356.0 ± 460.2	311.177	41
PGX	2058.6 ± 207.9	33	2818.2 ± 515.0	961.498	32
**Vitamin D (µg)**					
Control	3.9 ± 0.5	39	3.4 ± 0.3	-0.498	40
Psyllium	3.5 ± 0.3	41	3.2 ± 0.3	-0.380	41
PGX	3.7 ± 0.5	33	3.0 ± 0.3	-0.685	32
**Folate (µg)**					
Control	414.0 ± 35.6	39	340.3 ± 24.5^a^	-71.103	40
Psyllium	406.4 ± 32.8	41	340.7 ± 22.4^a^	-65.642	41
PGX	310.0 ± 19.5	33	320.5 ± 24.7	48.145	32
**Iodine (µg)**					
Control	135.6 ± 8.2	39	138.3 ± 9.2	2.792	40
Psyllium	125.4 ± 7.1	41	130.1 ± 8.2	4.754	41
PGX	118.8 ± 8.2	33	118.1 ± 7.6	-0.894	32
**Potassium (mg)**					
Control	3279.3 ± 122.3	39	3238.9 ± 181.4	-44.486	40
Psyllium	3134.1 ± 141.4	41	2905.0 ± 123.5	-229.108	41
PGX	2855.1 ± 134.3	33	2825.7 ± 142.1	36.487	32
**Sodium (mg)**					
Control	3081.846 ± 185.5	39	2844.8 ± 157.0	-214.018	40
Psyllium	2615.4 ± 188.0	41	2334.8 ± 128.5^b^	-280.569	41
PGX	2639.4 ± 179.1	33	2490.1 ± 150.0	-93.027	32
**Iron (mg)**					
Control	14.2 ± 0.8	39	12.9 ± 0.7	-1.389	40
Psyllium	12.0 ± 0.6	41	11.1 ± 0.6	-0.904	41
PGX	11.7 ± 0.7	33	11.6 ± 0.8	0.531	32
**Zinc (mg)**					
Control	14.7 ± 0.8	39	13.9 ± 0.8	-0.900	40
Psyllium	13.6 ± 1.0	41	11.8 ± 0.7^a^	-1.857	41
PGX	11.6 ± 0.7	33	12.7 ± 1.1	1.321	32
**Calcium (mg)**					
Control	952.3 ± 62.6	39	885.1 ± 56.0	-62.324	40
Psyllium	888.3 ± 50.3	41	806.0 ± 44.2	-82.305	41
PGX	834.3 ± 60.6	33	754.0 ± 57.3	-74.471	32
**Magnesium (mg)**					
Control	400.8 ± 22.0	39	369.9 ± 19.4	-31.998	40
Psyllium	371.9 ± 19.9	41	334.3 ± 15.8^a^	-37.584	41
PGX	359.4 ± 22.4	33	349.1 ± 23.8	-5.595	32

^*^Values are expressed as mean ± SEM. ^a^significant compared to baseline at *p* < 0.05; ^b^significant compared to Control at *p* < 0.05. Between-groups difference was examined using one-way ANOVA and within-group difference was examined using a paired samples t test

Table 4 refers to the macronutrient intake levels from self-recorded dietary intake from baseline to 3 months. There were significant changes at *p* < 0.05 from baseline for both psyllium and PGX® with a reduction in dietary energy intake following 3 months fibre supplementation. Psyllium decreased from 8859.4 ± 318.4 kJ/d to 7272.3 ± 218 kJ/d with a mean change of -1617.3 kJ/d. The PGX® group went from 8783.3 ± 261.3 kJ/d to 7556.3 ± 243.1 kJ/d with a mean reduction of -1090.9 kJ/d. Carbohydrate intake also significantly reduced in both fibre groups with psyllium the group going from 212 ± 8.9 g/d at baseline to 181 ± 7.5 g/d at 3 months (-31.8 g/d. PGX® also decreased from 209.8 g/d ± 8.6 of carbohydrate to 175.3 ± 8.4 g/d (-31.5 g/d). For fat intake, the psyllium group was the only significant change from baseline with 81.2 g/d ± 4.5 at week 0 and 66.9 g/d ± 3.1 at 12 weeks, with a mean change of -15.6 g/d. Protein intake also only reduced significantly in the psyllium group with 104 g/d ± 4.7 recorded at baseline and 85.9 g/d ± 3.6 after 3 months fibre supplementation (-17.6 g/d). Fibre intake for both fibre groups increased significantly from baseline to 3 months, with psyllium increasing 12.7 g/d from 23.9 g/d ± 1.3 to 36.4 g/d ± 1.2 and PGX® increasing 14.2 g/d from 21.4 g/d ± 1.2 to 36.6 g/d ± 1.3.

**Table 4:** Self-recorded dietary intake of macronutrients for baseline to 3 months of psyllium or PGX fibre supplementation

Variable	Group	Baseline	n	3 months	n	Mean change	*P*
Energy (kJ/d)	CTR	8636.4 ± 294.4	44	9013.1 ± 223.6^a^	39	115.5	0.641
	PSY	8859.4 ± 318.4	43	7272.3 ± 218^b^	41	-1617.3	0.000
	PGX	8783.3 ± 261.3	39	7556.3 ± 243.1^b^	33	-1090.9	0.001
CHO (g/d)	CTR	209.8 ± 8.8	44	212.6 ± 7.7^a^	39	-1.6	0.827
	PSY	212 ± 8.9	43	181 ± 7.5^b^	41	-31.8	0.002
	PGX	209.8 ± 8.6	39	175.3 ± 8.4^b^	33	-31.5	0.003
Fat (g/d)	CTR	80.2 ± 4.2	44	83.8 ± 3.2^a^	39	-0.5	0.907
	PSY	81.2 ± 4.5	43	66.9 ± 3.1^b^	41	-15.6	0.000
	PGX	82.8 ± 3.3	39	72.2 ± 3.4^b^	33	-9	0.055
Protein (g/d)	CTR	101.7 ± 4	44	109.9 ± 3.7^a^	39	6.4	0.169
	PSY	104 ± 4.7	43	85.9 ± 3.6^b^	41	-17.6	0.000
	PGX	101.6 ± 3.9	39	94.4 ± 4.1^b^	33	-6.5	0.174
Total Fibre (g/d)	CTR	22.6 ± 1.2	44	24.2 ± 1.2^a^	39	0.8	0.599
	PSY	23.9 ± 1.3	43	36.4 ± 1.2^b^	41	12.7	0.000
	PGX	21.4 ± 1.2	39	36.6 ± 1.3^b^	33	14.2	0.000

Values are mean ± SEM with baseline as a covariate. Mean change from baseline. *P* values are within group differences compared to baseline. Different letters in superscript represent significant differences between groups *p* < 0.05. Between-groups difference was examined using one-way ANCOVA. *CHO* (carbohydrate), *CTR* (control), *PGX* (PolyGlycopleX), *PSY* (psyllium)

## Discussion

Over the years, research has consistently shown that a higher intake of dietary fibre correlates with a reduced risk of several chronic diseases, including cardiovascular diseases (CVDs), cancer, type 2 diabetes, and obesity [11]. The health benefits provided by a higher dietary fibre intake are thought to occur via the following mechanisms: delaying the absorption of glucose; increased satiation and satiety effect which can reduce food intake, and may promote weight loss; gut microbe–induced production of short-chain fatty acids, which have immunomodulatory and anti-inflammatory properties; trapping of bile acids and carcinogenic substances; and increased intake of biologically active compounds, such as phytochemicals and antioxidants [28, 29]. However, it is still not well known how a higher fibre intake through supplementation affects micronutrients absorption in the gastrointestinal tract, especially in overweight and obese individuals. Therefore the aim of this study was to determine whether 3-months’ supplementation with psyllium or PGX® fibre affects micronutrient status of overweight and obese adults.

Current literature, although limited suggests that a higher fibre intake can either inhibit or promote bioavailability depending on the nutrient, the absorptive mechanism involved, and the composition of the food matrix as a whole [30]. Factors affecting vitamin or mineral bioavailability include how it is absorbed, excreted, stored/distributed; whether its sequestered by fat or dispersed in tissue, metabolic processes (catabolic losses, possibly oxidative), increased physiologic requirements, or lower absolute total dietary intake [31, 32]. This may help explain the variability that can occur within serum micronutrient levels. The habitual diet of overweight and obese individual is often seen as energy-dense and nutrient-poor, so maintaining nutritional sufficiency can potentially be compromised. An emerging theory suggests that nutritional deficiency states in obese persons are not more prevalent, as they may innately consume more food to compensate for the poor nutritional quality of their diet to achieve sufficiency. However due to the satiating effects of fibre, if a therapeutic dose is consumed, less food consumption may occur. Therefore nutrient intake and nutrient status may be affected in the long term, if malabsorption from the combined effects of obesity on bioavailability and a poor diet are already in effect.

This study examined the micronutrients present in serum samples and self-reported dietary intake data of overweight and obese individuals at baseline and 3 months following consumption of 15 g per day of either a control (rice flour), psyllium or PGX® fibre supplement. According to the serum values (Table 1 and Table 2), there were no between group differences in micronutrient status compared to control, both at baseline as well as after 3 months of fibre supplementation. Self-reported dietary intake using the 3-day food diaries (Table 1) showed the baseline value for zinc was significantly different in the PGX® group compared to the control group. As this is a baseline value, its significance is probably not related to an increase in dietary fibre, and could be explained by simple misreporting or poor zinc status in overweight and obese [33, 34]. All other micronutrients for self-reported dietary intake data at baseline, showed no significant differences compared to control at *p* < 0.05.

There was no significant difference between groups in serum micronutrient levels following 3 months fibre supplementation (Table 2), however there was a notable increase or decrease for some key micronutrients from baseline to 3 months (within groups) (Table 1 and Table 2). Folate, sodium and potassium all showed changes from baseline, while not significant at *p* < 0.05 it shows the potential for serum levels to be further affected over a longer time period (greater than 3 months). For between group differences in self-reported dietary nutrient intake after three months of fibre supplementation, sodium intake was found to be significantly lower in the psyllium group compared to the control (Table 3). Dietary intake data for vitamin C was also significantly lower in the PGX® group at 3 months compared to the control group at *p* < 0.05. Other micronutrients for dietary intake data were also lower following 3 months dietary fibre supplementation but not statistically significant at p < 0.05. These included iodine and calcium in the PGX® group compared to the psyllium group and control group (Table 3). There appeared to be a downward trend for most dietary micronutrients in both fibre groups at 3 months, compared to the control (Table 3).

While these results may reflect differences or changes in dietary intake (respectively), it may potentially be the result of inaccurate self-reporting [33]. It is well known that overweight and obese participants frequently under-report dietary intake in self-reported food diaries [35], therefore these results may be due to simple misreporting of 3-day food diaries completed by participants as opposed to any actual changes. Another possibility of reduced nutrient intake data after 3 months dietary fibre supplementation of 15 g per day is due to the satiating effect of dietary fibre. Participants physically consumed less food as they were simply not as hungry, meaning over all, recorded dietary macro and micronutrient intake was reduced. This theory was supported by the significant reductions in energy, fat, carbohydrate and protein and the significant increase in fibre intake per day in the self-recorded dietary macronutrient intake following 3 months fibre supplementation (Table 4).

The fact that there were no between group differences in the serum micronutrients following three months of PGX® or psyllium fibre supplementation is a positive outcome for the present study. Dietary fibre supplementation is becoming recognised as an adjunct to weight management programs for overweight and obese individuals, and potentially complements many existing weight loss programs. However, as with any new treatment, it is essential to eliminate any negative effects that a new method or product may have on patients’ health, in this instance their micronutrient status. Recent research promotes the safety and efficacy of the novel fibre supplement PGX®, as well as showing positive effects for metabolic syndrome (MS) and associated risk factors such as CVD, blood pressure and blood lipids [13, 27]. However the effect of PGX® or psyllium fibre on micronutrient status, when used as a part of a weight loss treatment in overweight and obese individuals, has not been well researched to date. The micronutrients examined in this study play an essential role in regulating biochemical pathways associated with metabolism. As obesity is a multifaceted and complex disease, the daily intake of 15 g of supplemented dietary fibre to help weight loss, needed to be ruled out as a possible confounder to attaining a better health outcome for this subpopulation.

### Strengths and limitations

This study is unique in that there has been very limited research examining the micronutrient status of overweight and obese individuals taking high fibre doses to assist with weight loss. While there is still much to learn about how fibre and micronutrients interact within the digestive system, the results of this study add to the body of knowledge in this area.

One of the limitations of this study was the time frame examined (baseline to 3 months only). It is possible that while no significant differences were found in micronutrient status following 3 months of high supplementary fibre (15 g per day), longer term fibre treatment or higher fibre dosages may have produced different results. It would be advisable for any future studies in this area to be conducted over a greater duration than 3 months as well as vary the dosage of dietary fibre supplement. A second limitation in this study was the use of self-reported dietary intakes. With any self- reporting nutritional data, potential bias is introduced as overweight and obese populations are most likely to under report their dietary intake [36]. While food diaries are a useful tool in the examination of dietary intake in weight management trials, the fluctuations in folate, zinc, magnesium sodium and vitamin C data found in this study may not represent changes to dietary intake, but rather inconsistent food consumption reporting. Thirdly the fibre dosage itself needs to be considered. Participants were consuming an extra 15 g of fibre each day in supplement form prior to breakfast, lunch and dinner. If this fibre amount was to increase to attempt to maximize or encourage further weight loss, this may produce more significant effects on serum micronutrient status. A further point to consider for any future studies is whether any significant results or differences between fibre groups from baseline to 3 months is due to the increase in PGX® or Psyllium fibre supplementation and not due to possible weight loss. High dietary fibre promotes satiety, so participants physically consumed less food and fewer kilojoules. Over a longer time period this possibly may have led to more significant differences in serum micronutrients. Another limitation was the lack of intention-to-treat analysis as the exclusion of dropouts from the study may have introduced bias.

### Significance

As high fibre diets have been linked to deficiencies of calcium, iron, trace metals, and certain vitamins in previous studies, it is important to update the body of knowledge in determining the possible health implications of therapeutic doses of fibre. If fibre is to be recommended as a potential adjunct to weight loss treatments for overweight and obese individuals, it is important to identify any possible limiting factors. The results from this research trial provide further evidence as to the physiological and biochemical effects high intakes (15 g daily) of psyllium or PGX® fibre supplementation have on the micronutrient status of an overweight or obese individual.

## Conclusions

Research examining methods to curb the obesity epidemic have focused on the development of safe and effective treatment options to assist with the current weight management strategies. One such option is the use of fibre supplements, which has been proven to improve satiety and reduce metabolic syndrome risk factors in overweight and obese individuals [13, 24]. Previous research had suggested that higher fibre consumption may impair the bioavailability of certain micronutrients, such that interference to the food matrix may influence the release, transformation, and subsequent absorption of some nutrients in the digestive tract [5]. This in turn may influence energy metabolism potentially contributing to and continuing the cycle of ongoing weight issues. As there were no significant changes in serum micronutrient status following 3 months of 15 g daily PGX® or psyllium fibre intervention, it would appear that short-term fibre supplementation may be adopted as a useful strategy to assist with weight management without impacting on the micronutrient status of overweight and obese individuals. However self-recorded dietary intake data of macro and micronutrient intake for this study illustrated that there is a downward trend in nutrient intake over 3 months due to the satiating effect of fibre. Thus, research over a longer study period as well as different fibre dosages is needed before this strategy can be confidently recommended as an ongoing treatment to aid weight loss in overweight and obese individuals.

## Supplementary Information

**Additional file 1.** (DOC 496 kb)

## Data Availability

The datasets used and/or analysed during the current study are available from the corresponding author on reasonable request.

## Abbreviations

PGX: PolyGlycopleX®
DF: Dietary fibre
BMI: Body mass index
BP: Blood pressure
CVD: Cardiovascular disease
HDL-C: High density lipoprotein cholesterol
LDL-C: Low density lipoprotein cholesterol
SCFA: Short-chain fatty acid
MS: Metabolic syndrome
NRVs: Nutrient reference values
NHMRC: National Health and Medical Research Council
RDIs: Recommended dietary intake
AI: Adequate intake
UL: Upper level
RBC: Red blood cell
TBW: Total body water
CNS: Central nervous system
CHD: Coronary heart disease
NIDDM: Non-insulin dependent diabetes mellitis
NSP: Non starch polysaccharide
DNA: Deoxyribose nucleic acid
ELISA: Enzyme-linked immunoabsorbant assay
ANCOVA: One way analysis of covariance
FAAS: Flame atomic absorption spectroscopy
SEM: Standard error of mean
AACB: Australasian Association of Clinical Biochemists
RE: Retinol equivalents
