# Supplementary material for: Micronutrient status of individuals with overweight and obesity following 3 months’ supplementation with PolyGlycopleX (PGX®) or psyllium: a randomized controlled trial
Source: BMC Nutr. 2022 May 3;8:42. doi: 10.1186/s40795-022-00534-7 (PMC9063372; doi:10.1186/s40795-022-00534-7)
Supplement: Supplementary file 1 — Additional file 1. (DOC 496 kb) [file 40795_2022_534_MOESM1_ESM.doc]

**APPENDIX 1: SCREENING SURVEY**

**SCREENING CHECKLIST - INCLUSION/EXCLUSION CRITERIA**

**OVERWEIGHT AND OBESE AUSTRALIAN ADULTS AND FIBRE SUPPLEMENTATION: ITS EFFECTS ON VITAMIN, MINERAL AND ANTIOXIDANT STATUS.**

Date:…………………

**Name:…………………………………………………………………………..**

**Address:………………………………………………………………………..**

**………………………………………………………………………………….**

**Phone No. (Circle best contact)**

**H:…………………………W:……………………………….Mobile:…………………………..**

**EMAIL……………………………………………………....** **DOB:…………… Age:……**

**Male/Female Height:…………… Weight:…………… Waist:………….. BMI:……**

| **Medical History** | **Yes** | **No** | **Details** |
| --- | --- | --- | --- |
| Are you a Smoker |  |  |  |
| Have you had your cholesterol, TG, glucose measured recently? Results? |  |  |  |
| Medications: – warfarin, steroids, thyroid, lipid lowering etc., what for? |  |  |  |
| Are you taking any nutritional supplements |  |  |  |
| Major operations |  |  |  |
| Any foods you can’t have? |  |  |  |
| GI disorders eg. Irritable bowel syndrome, coeliac disease, diarrhoea |  |  |  |
| Major illnesses/diseases: Do you have   - Diabetic - Kidney disease (renal problems) - Liver disease - Heart disease |  |  |  |
| Have you ever received a formal diagnosis by a professional for mental illness? |  |  |  |
| Do you have High Blood Pressure |  |  |  |
| Do you have asthma |  |  |  |
| Allergies (esp. Bee stings) |  |  |  |
| Do you play a sport or exercise regularly |  |  |  |
| Supervised weight loss programs? |  |  |  |
| Regularly have >2 alcoholic drinks/day |  |  |  |
| Difficulty taking blood? |  |  |  |
| Have you been involved in any other studies in the last 6 months? |  |  |  |

1. Are you able to get to Curtin University? Yes / No
2. Can we keep your contact details for future reference in clinical research? Yes / No


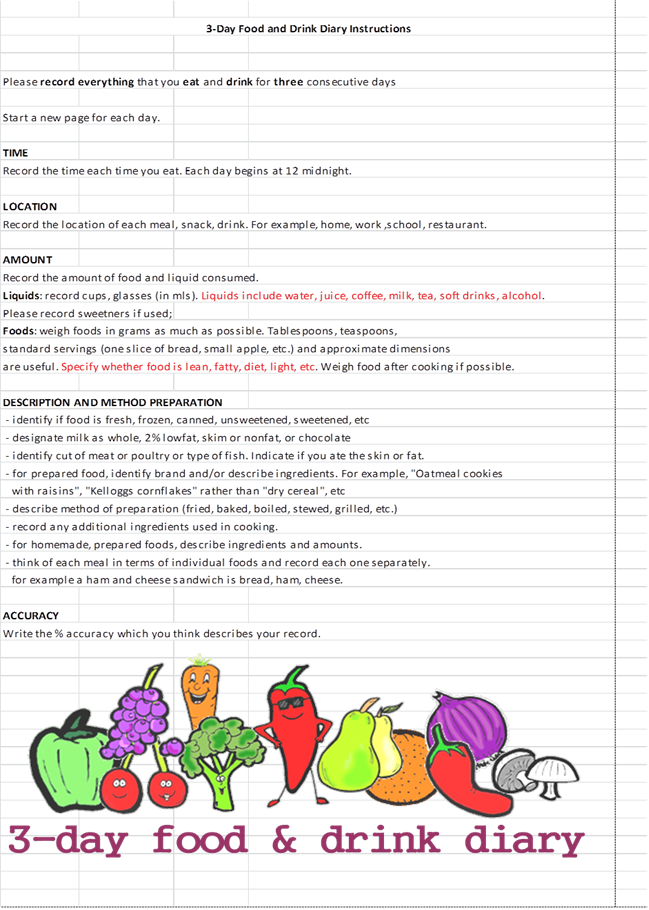
**APPENDIX 2: THREE-DAY FOOD DIARY**

**
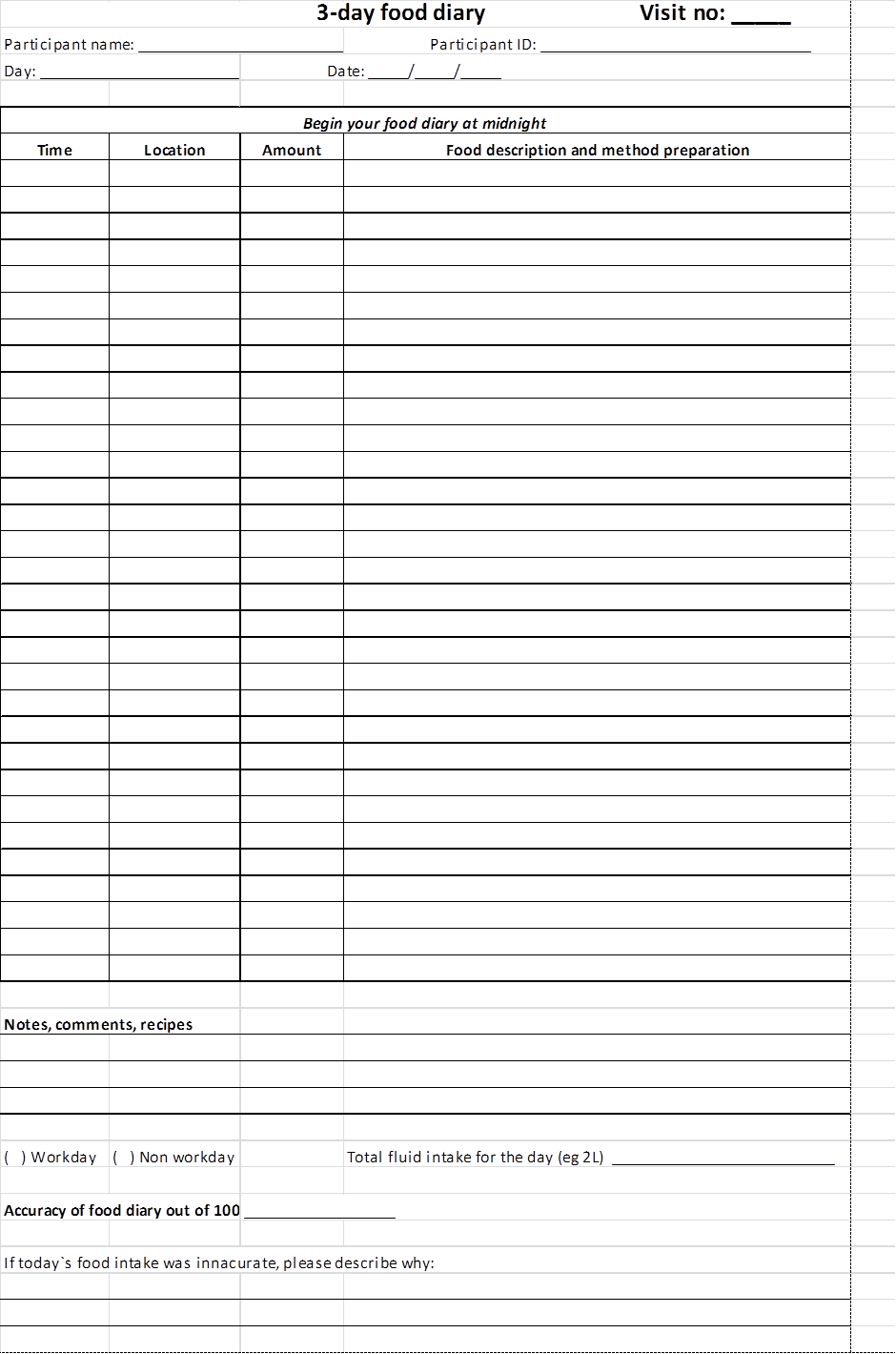
**

**APPENDIX 3: SUPPLEMENT INTAKE CALENDAR**

**Please tick off each box after each meal

**If you miss a fibre supplement, don't tick the box

Bring your completed calendar to your next long clinical visit

Long Clinical Visit Date: _____
